# Reproductive success of captively bred and naturally spawned Chinook salmon colonizing newly accessible habitat

**DOI:** 10.1111/j.1752-4571.2012.00271.x

**Published:** 2012-06-11

**Authors:** Joseph H Anderson, Paul L Faulds, William I Atlas, Thomas P Quinn

**Affiliations:** 1 School of Aquatic and Fishery Sciences, University of WashingtonSeattle, WA, USA; 2 Seattle Public UtilitiesSeattle, WA, USA

**Keywords:** conservation, dams, hatchery, natural selection, pedigree, reintroduction, sexual selection

## Abstract

Captively reared animals can provide an immediate demographic boost in reintroduction programs, but may also reduce the fitness of colonizing populations. Construction of a fish passage facility at Landsburg Diversion Dam on the Cedar River, WA, USA, provided a unique opportunity to explore this trade-off. We thoroughly sampled adult Chinook salmon (*Oncorhynchus tshawytscha*) at the onset of colonization (2003–2009), constructed a pedigree from genotypes at 10 microsatellite loci, and calculated reproductive success (RS) as the total number of returning adult offspring. Hatchery males were consistently but not significantly less productive than naturally spawned males (range in relative RS: 0.70–0.90), but the pattern for females varied between years. The sex ratio was heavily biased toward males; therefore, inclusion of the hatchery males increased the risk of a genetic fitness cost with little demographic benefit. Measurements of natural selection indicated that larger salmon had higher RS than smaller fish. Fish that arrived early to the spawning grounds tended to be more productive than later fish, although in some years, RS was maximized at intermediate dates. Our results underscore the importance of natural and sexual selection in promoting adaptation during reintroductions.

## Introduction

The field of reintroduction biology aims to understand the ecological, demographic, and genetic factors that lead to establishment of self-sustaining populations in areas where they had been extirpated (Seddon et al. 2007). In general, programs that release many individuals and those that use primarily wild source populations tend to be more successful (Wolf et al. 1996; Fischer and Lindenmayer 2000). The use of captively bred animals, therefore, represents a difficult trade-off for resource managers. Captive breeding can increase the initial abundance of colonists if wild animals are not available or are difficult to transplant, but it also carries certain genetic risks that may affect long-term sustainability.

One of the primary risks is that captive-born individuals will have lower fitness than wild animals. Examples from reintroduced birds and mammals indicate that captively bred individuals exhibit lower performance at fitness-related traits such as survival and reproductive success (RS) than wild-born animals (Brown et al. 2006; Jule et al. 2008; Roche et al. 2008; Aaltonen et al. 2009; Evans et al. 2009). Although this could occur via several mechanisms, domestication selection in the captive environment often reduces the fitness of animals for life in the wild and can profoundly decrease the likelihood of reintroduction success (Frankham 2008). Maintaining natural rather than artificial patterns of selection might be particularly important during reintroduction because colonization of new environments offers special opportunities for adaptive evolution that can increase the likelihood of establishment and persistence (Reznick and Ghalambor 2001; Lambrinos 2004; Kinnison and Hairston 2007). If selection on the traits of colonizers is common, it would suggest that promoting natural evolutionary processes should be considered in planning and implementing reintroductions.

Whether or not to use captively bred animals in reintroduction programs is a pressing issue for Pacific salmon (*Oncorhynchus* spp.). Hatcheries are pervasive throughout the native range of salmon (reviewed by Fraser 2008; Kostow 2009; Naish et al. 2008); therefore, most managers planning reintroductions would have access to artificial supplementation facilities. Captively reared salmon can provide an immediate demographic boost to populations targeted for reintroduction or conservation-oriented enhancement (Berejikian et al. 2008). On the contrary, inclusion of hatchery fish may depress overall population productivity (Chilcote et al. 2011). Hatchery fish, especially those from nonlocal sources, tend to have lower RS than wild fish when both groups breed in sympatry (reviewed by Araki et al. 2008). Although the precise mechanisms remain unclear, such fitness declines have been observed after as little as one or two generations in captivity (Araki et al. 2007).

Impassable dams and culverts prevent salmon from reaching historically accessible spawning and rearing habitats in many rivers (National Research Council 1996), and restoration of migratory corridors is an important conservation strategy. Despite their homing ability (Quinn 2005), salmon naturally colonize new habitats (Milner et al. 2000; Quinn et al. 2001; Ciancio et al. 2005; Anderson and Quinn 2007). Such dispersal may obviate the need for directed salmon transplantation or hatchery supplementation following the removal of migration barriers, particularly if there are nearby source populations. Even if supplementation is not necessary for successful colonization in the long term, there is pressure to use hatchery salmon to accelerate the rate of population expansion (Young 1999). Agencies removing barriers are therefore confronted with difficult decisions in the management of recolonizing salmon populations. Should hatchery fish be used to increase the rate of recolonization? If so, how many and at which life stage (juvenile, adult, etc.) should they be planted? If not, should hatchery fish that naturally stray into the new habitat be allowed to spawn there or be culled? Should hatchery fish be allowed only in the initial stages of recolonization, and if so, at what point would they no longer be needed?

In addition to hatchery versus wild origin, individual traits such as body size and the timing of breeding are likely to affect the RS of salmon colonists. In both sexes, larger fish typically produce more offspring (Seamons et al. 2007; Ford et al. 2008; Anderson et al. 2010; Williamson et al. 2010), a trend that is often attributed to size-biased competition for breeding resources (Fleming and Gross 1994; van den Berghe and Gross 1989). The relationship between RS and the timing of reproduction has been less consistent than body size, as studies have found that selection favored earlier salmon (Williamson et al. 2010), favored later salmon (Ford et al. 2008), or showed substantial variation in direction and shape among years and sexes (Dickerson et al. 2005; Seamons et al. 2007; Anderson et al. 2010). This variance is likely related to the temporally dynamic river conditions encountered by breeding adults and their offspring. Measuring natural selection on individual traits during colonization provides crucial information for designing effective reintroduction programs (Hendry et al. 2003).

In this work, we address the role of captively bred animals during reintroduction by using, as an example, a population of Chinook salmon, *O. tshawytscha* (Walbaum, 1792), in the Cedar River, WA. Modification of Landsburg Diversion Dam in 2003 made 33 km of spawning and rearing habitat accessible for the first time in over a century. Chinook salmon are listed as threatened in this region under the Endangered Species Act, and thus are of particular conservation concern. Hatchery fish were not transplanted above the dam, but adults were allowed to bypass the dam and spawn if they volitionally entered the fish passage facility. We sampled these colonizing Chinook salmon in 2003–2009 and used molecular DNA markers to evaluate the RS of hatchery and naturally spawned salmon. Our analysis had three primary objectives. First, we evaluated the demographic benefit of permitting the hatchery fish to spawn by quantifying the number of hatchery origin colonists and their cumulative numerical contribution to the next generation of the expanding population. Second, we compared per capita RS between hatchery and naturally spawned fish to evaluate the potential for a fitness cost associated with colonization by hatchery fish. As a result of previous and current gene flow between local hatcheries and Chinook salmon spawning naturally in the Cedar River, this comparison essentially tested the effects of one generation in the hatchery. Finally, to assess mechanisms for any observed fitness differences and evaluate the capacity for adaptive evolution during reintroduction, we measured selection on body size and date of arrival to the spawning grounds.

## Methods

### Study site natural history and sampling

**Figure 1:**
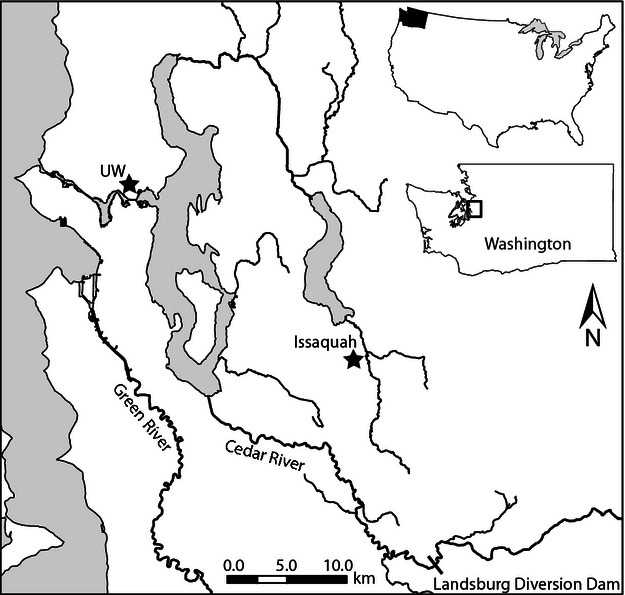
Cedar River and Lake Washington basin. The two hatcheries in the area producing Chinook salmon are denoted by stars. Chinook salmon have been observed spawning in each of the tributaries shown on the map. Modified from Pess et al. (2011).

The Cedar River flows west from the Cascade mountain range into the south end of Lake Washington, which is connected to Puget Sound via a man-made shipping canal through Seattle, Washington, USA (Fig. 1). Chinook salmon in the Lake Washington basin have a complicated natural history owing to hydrologic changes and hatchery transfers into the watershed, both of which affected connectivity with the neighboring Green River. Historically, the Cedar River was connected to Puget Sound via the Green and Black rivers, although the extent to which the Cedar River Chinook salmon were distinct from those in the Green River remains unclear (Ruckelshaus et al. 2006). In 1916, the Cedar River was diverted into Lake Washington in conjunction with construction of the shipping canal and navigational locks. Chinook salmon from a hatchery on the Green River founded the Issaquah Creek hatchery population (Fig. 1) in 1937 and continued to supply broodstock until 1992 (HSRG 2003). The Issaquah Creek hatchery is the primary production facility in the basin, spawning approximately 2500 of the 3069–13 482 adult Chinook salmon trapped in 2003–2009 (Hatchery Escapement Reports, Washington Department of Fish and Wildlife, http://wdfw.wa.gov/hatcheries/escapement). The only other Chinook salmon hatchery in the basin is a smaller facility operated by the University of Washington (UW) that was founded with Green River origin Chinook salmon in 1949 (HSRG 2003). At this hatchery, approximately 250 of the 1187–2738 returning adults in 2003–2009 were spawned (J. Wittouck, hatchery manager, personal communication).

There is no Chinook salmon hatchery on the Cedar River, but hatchery fish routinely spawn there. Hatcheries in this area typically excise the adipose fin of juveniles prior to release, and approximately 28–34% of adult Chinook salmon sampled in the Cedar River below Landsburg Diversion Dam in 2003–2005 were produced in hatcheries (Berge et al. 2006). Furthermore, recent analysis found little genetic differentiation between fish collected at the Issaquah Creek hatchery and naturally spawned fish on the Cedar River breeding grounds (*F*_ST_ = 0.001–0.002) (Warheit and Bettles 2005). The data from marked fish and genetic analysis both indicated straying from the basin's hatcheries into the naturally spawning population of the Cedar River. In our study, unmarked fish (i.e., intact adipose fin) may have had recent hatchery ancestry, so we use the term ‘naturally spawned’ rather than ‘wild’ to indicate that our knowledge of each individual's background extends only to the previous generation.

Landsburg Diversion Dam, at river km 35.1, blocked fish migration from 1901 to fall 2003, when modifications to the dam enabled salmon to recolonize approximately 33 km of habitat above the dam on their own volition. There was no active transplantation or hatchery supplementation. For the vast majority of the migration period, the fish ladder was configured such that adult salmon could not bypass the dam without being handled by staff, allowing us to sample virtually all colonists. A few salmon ascended without being sampled, but an automatic camera system (described by Shardlow and Hyatt 2004) indicated that > 98% of the Chinook salmon were sampled in 2003, 2005, and 2007–2009; equipment malfunction precluded assessment in 2004 and 2006. We identified each sampled fish by species and sex, measured fork length, recorded the date of dam passage (i.e., date of arrival to the spawning grounds), and took a small tissue sample for DNA analysis.

Hatchery fish were identified by a missing adipose fin. The Issaquah Creek hatchery did not mark Chinook salmon prior to 1999; therefore, any 5-year-old salmon in 2003 with adipose fins might have been hatchery produced. However, parentage analysis (see Results section) indicated that very few salmon were this old. From 1999 to 2007, both hatcheries in the basin marked the vast majority of released Chinook salmon (pooled estimate: range = 94.0–99.3%, median = 97.9%, Regional Mark Processing Center, http://www.rmpc.org). An important component of hatchery operations is whether they incorporate naturally spawned fish into broodstock under an integrated model or breed exclusively hatchery fish under a segregated model (Mobrand et al. 2005). For much of its history, the Issaquah hatchery did not mass mark releases, and bred some naturally spawned fish because they could not be distinguished from the hatchery fish. Regardless, any wild population that historically inhabited Issaquah Creek has been extirpated; therefore, the current population (including naturally spawned fish) are descendants from the Green River hatchery transfers (Ruckelshaus et al. 2006). There is no natural reproduction associated with the UW hatchery, so it operates under a segregated model. Both hatcheries typically release juvenile Chinook salmon at age 0 in late spring, several months after hatching, when most naturally spawned fish would be migrating to sea.

We genotyped samples at 10 microsatellite loci with standard protocols: *Omm1046, Omm1080, Omm1130, Omm1241, OtsG68, Ots201, Ots208, Ots209, Ots212,* and *Ots527*. Primer sequences and polymerase chain reaction (PCR) protocols are described by Anderson (2011). Microsatellite PCR products were size-fractionated and visualized on a MegaBACE 1000 automatic genotyper (GE Healthcare, Piscataway, NJ, USA); genotypes were assigned using Genetic Profiler version 2.2.

All individuals described here (*n* = 1052) were genotyped at ≥7 loci, and 98.9% were genotyped at ≥9 loci. Across all return years 2003–2009, we obtained insufficient genetic data (i.e., <7 loci genotyped) from five samples, and these fish were excluded from all analyses. To quantify our genotyping error rate, we re-extracted DNA from 145 samples and genotyped them again as a positive control, including one individual on each 96-well plate of genomic DNA used in the study. These samples provided 2680 single locus genotypes which were independently amplified and scored from at least two different sources of genomic DNA. Only 0.56% of these genotypes conflicted with another from the same sample, and we consider this to be our error rate resulting from mishandling of samples, scoring mistakes, or other human errors. Cervus version 3.0.3 estimated that the frequency of null alleles for each of the 10 loci was ≤0.021.

### Data analysis

*F*_STAT_ version 2.9.3.2 (Goudet 1995) was used to calculate Weir and Cockerham's (1984) pairwise *F*_ST_ for hatchery versus natural origin fish and the probability of population differentiation using the log-likelihood-based randomization test. Parentage analysis was used to quantify the demographic contribution of the hatchery salmon and compare their RS to that of the naturally spawned fish. We used Cervus version 3.0.3 (Marshall et al. 1998; Kalinowski et al. 2007), which assigns parentage based on a likelihood ratio (or LOD) score, for all parentage assignments. For analysis of the parental cohort that spawned in year x, all naturally spawned salmon sampled in years x+2, x+3, x+4, and x+5 were considered as potential offspring, with the constraint that 2009 was the final year of offspring sampling. The LOD threshold for assigning parentage was readily apparent by inspection of the LOD scores for the most likely parents for each potential offspring. We first considered mother–father–offspring trios, and the most likely trio for each offspring showed distinct non-overlapping modes of LOD scores: one where each trio had ≥3 mismatching loci (LOD: median = −10.6, range = −26.3 to 12.8) and one where each trio had ≤2 mismatching loci (LOD: median = 39.4, range = 22.4–48.4). Parentage was assigned for all offspring in the latter mode but none in the former. LOD scores for the most likely single parent for each offspring also showed two distinct modes: one ranging from −17.3 to 9.46 (median = −5.08) with 1–6 mismatching loci, and another ranging from 10.6 to 23.3 (median = 17.9) with 0–1 mismatching locus. Single parents were assigned to all offspring in the upper mode if they had not already been assigned both a mother and a father.

Several lines of evidence suggested that our parentage results were robust. LOD thresholds for both two and single parent assignments exceeded the 99% confidence level established through simulation within Cervus. Furthermore, for the vast majority of potential offspring that were not assigned to any parents, the most likely single parent had many mismatching loci (median = 4). Thus, not assigning parentage did not result from failure to distinguish between two equally likely parents, but rather because there was no potential parent in the sampling pool. Finally, we explored Colony (Jones and Wang 2010) as an alternative method of parentage assignment and found virtually identical results (average = 98.7% identical across four runs).

Reproductive success was defined as the total number of returning adult offspring produced by each spawning salmon. We had complete RS data (i.e., 2–5-year-old offspring) for the 2003 and 2004 cohorts. Data for the 2005 cohort did not include 5-year-old offspring, but we included it in our analysis because there were so few 5-year-old offspring in the other cohorts. Parentage assignment errors can bias the relative RS of two groups, such as the hatchery and naturally spawned fish in our study. Araki and Blouin (2005) demonstrated the importance of minimizing the rate of assignment to an untrue parent when the true parent is absent from the data set. We calculated this error rate by considering hatchery fish, whose parents were not sampled, as offspring for each parental cohort 2003–2005. Of 267 hatchery fish, one matched the criteria for the natural offspring assigned two parents, one was assigned a mother only, and five were assigned only fathers. These low error rates (males = 2.2%, females = 0.4%) suggest minimal bias in our estimates of relative RS. Furthermore, to avoid bias in relative RS estimates as a result of the ‘aunt and uncle effect,’ we made parentage assignments based on absolute LOD score rather than a threshold relative to the next most likely parent (Ford and Williamson 2010).

To test the null hypothesis that RS was similar for hatchery and naturally spawned fish, we used a generalized linear model (GLM) with negative binomial error structure and a log link function fit separately for each sex using the package MASS in the program R (Venables and Ripley 2002). The form of the model was RS = year + origin + year × origin, with the origin term simply indicating whether or not each fish was produced in a hatchery. The significance of year, origin, and their interaction was evaluated via analysis of deviance. We report the effect size as a result of origin as *δ* = *e*^*β*^, where *β* is the parameter estimate of the origin term, noting the log link in the model. Rather than estimating the power of our test on the origin term, we used the 95% confidence interval on *β* to provide the maximum effect size (*δ*_*max*_) such that we could reject the hypothesis that *δ* > *δ*_*max*_ following Colegrave and Ruxton (2003).

Natural selection was measured using standardized methods developed by Lande and Arnold (1983). Importantly, our lifetime RS data allowed us to measure natural selection directly rather than using a common fitness surrogate such as survival or number of juvenile offspring. Gradients measure direct selection on a trait independent of indirect selection resulting from the effects of a correlated measured trait, whereas differentials measure the combined effects of direct and indirect selection (Brodie et al. 1995). Fitness, defined as the total number of returning adult offspring, was converted to relative fitness (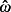) by dividing each individual's RS by the population mean within each season-sex. Body size (*s*) and date of arrival to the spawning grounds (*d*) were standardized by subtracting the season-sex mean and dividing by the standard deviation. Both gradients and differentials were calculated as regression coefficients fit by ordinary least squares, with 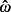 as the response variable. A single regression with both *s* and *d* as predictors gave the linear gradients; separate regressions with either *s* or *d* as predictors provided the linear differentials. Similarly, a single regression with *s*^2^, *s*, *d*^2^, *d*, and *s* × *d* as predictors gave the nonlinear gradients; separate regressions with *s*^2^ and *s* or *d*^2^ and *d* gave the nonlinear differentials. Nonlinear gradients and differentials were calculated as two times the quadratic regression coefficients.

To visualize the selection surface, we plotted cubic splines of raw RS against trait values using the gam function in R. The cubic splines used a negative binomial error structure, with the dispersion parameter *θ* estimated as [mean (RS)]^2^/[var (RS) – mean (RS)]. To avoid excessively fine-scaled cubic splines, we constrained the smoothing parameter λ to values ≥4.

## Results

Counts of Chinook salmon ascending the fish ladder at Landsburg Diversion Dam varied among years but tended to increase (Table 1). More males than females ascended in all years, and in most years, >75% of the salmon were male (Table 1). The proportion of hatchery fish tended to decrease over time, from approximately two-thirds of the original colonists in 2003 and 2004, to 17–30% in 2007–2009 (Table 1).

**Table 1:** Counts of adult Chinook salmon, both hatchery (H) and naturally spawned (N) origin, sampled and genotyped at Landsburg Diversion Dam on the Cedar River, Washington, USA

	Naturally spawned		Hatchery			Percentage		H versus N differentiation	
					
Year	Males	Females	Males	Females	Total	Hatchery %	Male %	*F*_ST_	P*
2003	18	6	42	10	76	68	79	0.0004	ns
2004	10	7	19	15	51	67	57	−0.0030	ns
2005	28	12	23	4	67	40	76	0.0037	ns
2006	79	20	69	12	180	45	82	0.0024	0.0088
2007	223	79	73	20	395	24	75	0.0021	<0.0001
2008	82	39	14	11	146	17	66	0.0021	0.086
2009	72	24	35	6	137	30	78	0.00026	0.0091
Pooled	512	187	275	78	1052	34	75	0.0008	<0.0001

* *P-*value from log-likelihood based randomization test, ns indicates *P* > 0.10.

Pairwise *F*_ST_ values showed weak differentiation, as a maximum of 0.37% of the genetic variation was attributable to differences between hatchery and naturally spawned fish within any 1 year, and a pooled sample across all years gave an *F*_ST_ of 0.0008 (Table 1). However, the randomization test indicated a significant (*P* < 0.05) difference in allele frequencies between hatchery and naturally spawned Chinook salmon in 2006, 2007, 2009, and the pooled estimate (Table 1). These comparisons tended to have a higher sample size (Table 1) and thus greater power to detect small differences in genotypes.

We assigned 225 adult Chinook salmon from 2006 to 2009 to parents that had bypassed the dam in 2003–2005. Most were assigned both a mother and a father (*N* = 149); mother-only assignments (*N* = 41) were slightly more common than father-only assignments (*N* = 35). Among the offspring from the two cohorts for which we had complete age structure, 9.3% were age-2, 53.5% were age-3, 36.0% were age-4, and 1.2% were age-5. Hatchery salmon made a significant demographic contribution to the next generation: 63.2% of the fish assigned mothers had hatchery mothers, 55.4% of the fish assigned fathers had hatchery fathers, and 36.9% of the fish assigned two parents had hatchery mothers and fathers.

**Figure 2:**
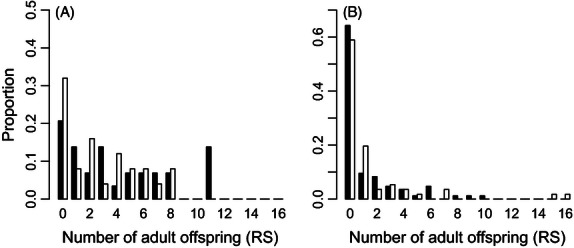
Reproductive success of hatchery (black) and naturally spawned (white) Chinook salmon for (A) females and (B) males pooled across years 2003–2005

Reproductive success varied greatly among individual salmon; many fish (especially males) produced no returning adult offspring, and a few produced many offspring (Fig. 2). Variance in RS, expressed as the opportunity for selection *I*, was greater in males than females (Table 2). Hatchery males had lower RS than naturally spawned males in all 3 years (range in relative RS = 0.70–0.90; Table 2). However, the GLM did not detect a significant difference in RS of hatchery versus naturally spawned males (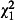 = 0.43, *P* = 0.51, *δ* = 1.43, *δ*_max_ = 4.46), although there was a difference between the years (year: 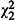 = 9.1, *P* = 0.012; origin × year: 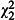 = 0.12, *P* = 0.94). There was no consistent trend in the relative RS of females, being higher for hatchery fish 2 years, and naturally spawned fish in 1 year. Mean RS across all years pooled was greater for hatchery females (Table 2), but the GLM predicted nonsignificantly higher RS for naturally spawned females (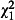 = 0.35, *P* = 0.55, *δ* = 1.39, *δ*_max_ = 4.21). As with males, female RS differed among years (year: 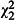 = 6.8, *P* = 0.033; origin × year: 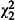 = 1.9, *P* = 0.38).

**Table 2:** Reproductive success (RS) comparison between hatchery and naturally spawned Chinook salmon. Rel RS is the mean RS of the hatchery fish divided by the mean RS the natural origin fish. *I* is the opportunity for selection (var/mean^2^). Sample sizes are contained in Table 1

		Hatchery RS		Natural RS		H + N pooled RS			
					
Sex	Year	Mean	Var	Mean	Var	Mean	Var	*I*	Rel RS
Males	2003	0.738	3.32	1.06	4.06	0.833	3.50	5.04	0.699
	2004	2.84	9.25	4.00	38.7	3.24	18.7	1.78	0.711
	2005	0.739	1.93	0.821	2.45	0.784	2.17	3.53	0.900
	Pooled	1.21	4.94	1.46	10.2	1.31	7.01	4.06	0.829
Females	2003	1.80	3.96	2.50	6.30	2.06	4.60	1.08	0.720
	2004	5.47	13.8	3.71	10.2	4.91	12.8	0.533	1.47
	2005	5.00	28.7	2.42	6.99	3.06	12.2	1.30	2.07
	Pooled	4.14	14.3	2.80	7.42	3.52	11.3	0.917	1.48

**Figure 3:**
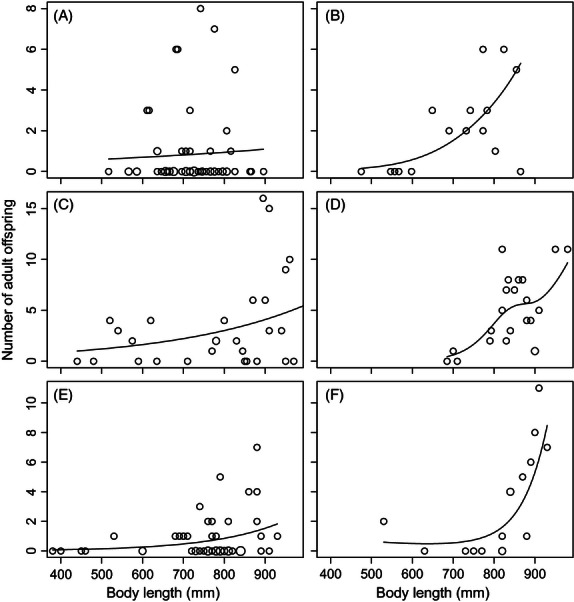
Relationship between body size and reproductive success (RS) for male (A, C, E) and female (B, D, F) Chinook salmon spawning in 2003 (A and B), 2004, (C and D), and 2005 (E and F). Lines represent cubic splines fit with a negative binomial error structure. In some cases, there are multiple observations on the same data point, and the size of the circle is proportional to the number of observations (maximum of four observations per point). One outlier male from 2004 (1100 mm, RS = 1) is not shown.

Larger salmon of both sexes tended to produce more adult offspring (Fig. 3). Linear selection coefficients (both gradients and differentials) on female body size were statistically significant in all 3 years and greater than those observed in males within each year (Table 3). In males, linear selection on body size increased in each subsequent year but was never statistically significant (Table 3). Males producing zero offspring were distributed throughout the range of sizes, including some very large males (Fig. 3). Statistically significant quadratic coefficients suggested nonlinear selection for the 2003 males and 2005 females based on Table 3, but the cubic splines did not corroborate this conclusion (Fig. 3A,F). For both sexes, heteroscedastic plots indicated that there was greater variance in RS at the larger sizes (Fig. 3). The selection gradients were largely similar to the differentials in sign and magnitude (Table 3).

**Table 3:** Selection gradients and differentials for body size and arrival date, with standard error in parentheses. Sample sizes are contained in Table 1

			Linear		Quadratic	
				
Type	Sex	Year	Size	Date	Size^2^	Date^2^
Gradients	Males	2003	0.0146 (0.285)	−0.672* (0.285)	−1.02* (0.216)	1.27* (0.243)
		2004	0.398 (0.247)	−0.192 (0.247)	−0.110 (0.281)	−0.549 (0.199)
		2005	0.492 (0.270)	−0.0710 (0.270)	0.490 (0.221)	0.485 (0.150)
	Females	2003	0.749* (0.265)	−0.205 (0.265)	−0.543 (0.364)	0.312 (0.322)
		2004	0.422^4^ (0.136)	−0.0678 (0.136)	0.113 (0.138)	−0.123 (0.106)
		2005	0.810* (0.362)	−0.196 (0.362)	2.55** (0.348)	1.04 (0.381)
Differentials	Males	2003	0.0898 (0.294)	−0.674* (0.281)	−0.342 (0.220)	1.00* (0.244)
		2004	0.404 (0.245)	−0.205 (0.254)	−0.105 (0.251)	−0.595 (0.193)
		2005	0.475 (0.260)	0.0478 (0.268)	0.401 (0.199)	0.262 (0.135)
	Females	2003	0.638* (0.219)	0.202 (0.273)	−0.374 (0.271)	−0.331 (0.232)
		2004	0.422** (0.133)	−0.0699 (0.163)	−0.0164 (0.106)	−0.230 (0.0710)
		2005	0.670* (0.247)	0.384 (0.287)	1.23*** (0.143)	0.353 (0.201)

*P*-value from *t*-test for significance of predictor.

* *P* < 0.05.

** *P* < 0.01.

*** *P* < 0.001.

**Figure 4:**
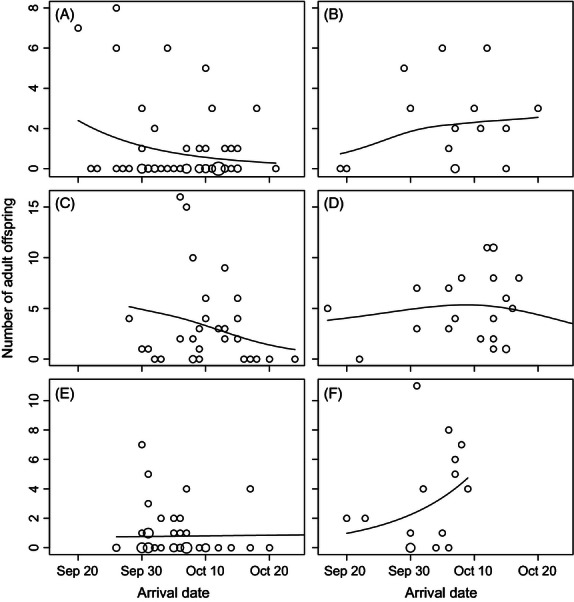
Relationship between arrival date and reproductive success (RS) for male (A, C, E) and female (B, D, F) Chinook salmon spawning in 2003 (A and B), 2004, (C and D), and 2005 (E and F). Lines represent cubic splines fit with a negative binomial error structure. In some cases, there are multiple observations on the same data point, and the size of the circle is proportional to the number of observations (maximum of nine observations per point). One outlier male from 2005 (November 4, RS = 2) and one female from 2004 (November 16, RS = 0) are not shown.

Patterns of selection on arrival date were less consistent than body size. The linear gradients, which were negative in all years, indicated an advantage to early arrival (Table 3). However, this relationship was only statistically significant for 2003 males and was negligible for the 2005 males and 2004 females. For the 2003 females and 2005 females, arrival date gradients differed substantially from the differentials, notably in sign (Table 3). In both these years, larger females arrived significantly later (Table 4), a likely explanation for the discrepancy. Except for the 2003 males, linear selection on arrival date was weaker than that on body size (Table 3). Despite the consistently negative linear gradients, in some years the individuals with the greatest RS had intermediate arrival dates (Fig. 4). However, only the 2004 females yielded a dome-shaped spline typical of stabilizing selection (Fig. 4). Although the positive quadratic gradient for the 2003 males suggested disruptive selection on arrival date, the cubic splines indicated that the relationship was more linear than quadratic (Fig. 4A).

**Table 4:** Pearson's correlation coefficient (*r*) between body size and arrival date, calculated from a sample pooled across both hatchery and naturally spawned Chinook salmon

Sex	Season	Correlation (*r*)
Males	2003	−0.112
	2004	−0.0319
	2005	0.241
Females	2003	0.543*
	2004	−0.00480
	2005	0.716**

* *P* < 0.05.

** *P* < 0.01.

***P < 0.001.

There were no statistically significant differences in body size or arrival date between hatchery and naturally spawned Chinook salmon for the three cohorts whose RS was measured. In both sexes, hatchery fish were slightly larger than naturally spawned fish when the body size data were pooled across seasons, but there was no consistent difference among years (Table 5). Two-way anovas, performed separately for each sex, indicated that size differed significantly between seasons (males: *F*_2,134_ = 6.19, *P* = 0.0027; females: *F*_2,48_ = 7.38, *P* = 0.0016) but not hatchery versus naturally spawned fish (males: *F*_1,134_ = 2.19, *P* = 0.14; females: *F*_1,48_ = 0.016, *P* = 0.90), and there was no interaction between the season and origin (males: *F*_2,134_ = 2.52, *P* = 0.085; females: *F*_2,48_ = 0.39, *P* = 0.68). On average, hatchery fish arrived later than naturally spawned fish in all years for both sexes (Table 5), but this difference was small (1–6 days) and not statistically significant (two-way anova, males: *F*_1,134_ = 0.51, *P* = 0.48; females: *F*_1,48_ = 0.066, *P* = 0.80). There was no detectable interaction effect (season × origin) on arrival date (males: *F*_2,134_ = 1.46, *P* = 0.24; females: *F*_2,48_ = 0.19, *P* = 0.83); males (*F*_2,134_ = 3.16, *P* = 0.046) but not females (*F*_2,48_ = 1.76, *P* = 0.18) differed significantly between seasons.

**Table 5:** Body size and date of arrival comparison between hatchery and naturally spawned Chinook salmon

		Mean length ± SD (mm)		Mean date ± SD (day)	
			
Sex	Year	Hatchery	Natural	Hatchery	Natural
Male	2003	700.6 ± 73.3	749.9 ± 82.6	October 7 ± 6.6	October 5 ± 7.6
	2004	802.6 ± 146.9	760.5 ± 213.5	October 11 ± 4.4	October 7 ± 8.1
	2005	779.6 ± 77.5	730.4 ± 149.9	October 8 ± 7.8	October 2 ± 4.2
	Pooled	745.3 ± 104.9	742.0 ± 144.0	October 8 ± 6.7	October 4 ± 6.4
Female	2003	699.6 ± 126.2	706.2 ± 126.0	October 6 ± 8.1	October 5 ± 9.8
	2004	851.3 ± 75.8	821.9 ± 73.7	October 12 ± 12.2	October 7 ± 8.5
	2005	855.0 ± 84.3	792.5 ± 112.3	October 4 ± 2.4	October 2 ± 5.2
	Pooled	799.5 ± 119.1	780.0 ± 111.3	October 9 ± 10.3	October 4 ± 7.5

## Discussion

This study explored the role of captively bred Chinook salmon during reintroduction, and evaluated the trade-off between the demographic boost versus the genetic risk of permitting them to spawn above a former migration barrier on the Cedar River, WA. Chinook salmon, including those produced in regional hatcheries, were permitted to colonize the new habitat if they arrived there volitionally. We first discuss our three analytical objectives in the context of salmonid evolutionary ecology and then consider the broader implications of our results to reintroduction programs.

### Reproductive success of hatchery and naturally spawned colonists

Our first objective was to quantify the demographic benefit provided by the hatchery fish, and they comprised a large fraction of the colonizing fish. In the first 3 years (when all salmon bypassing the dam were colonists), ≥40% of all Chinook salmon originated from a hatchery. However, the expanding population was almost certainly limited by the gametes provided by female salmon because they were scarce relative to the 33 km of available spawning habitat. Allowing hatchery fish above the dam doubled the number of spawning females that reached the new habitat in 2003 and 2004. Indeed, most of the salmon from 2006 to 2009 that had mothers assigned (120 of 190, or 63%) were produced by hatchery females. Many of these offspring probably would not have returned to the newly accessible habitat had their parents been denied entry to the fish ladder. Thus, permitting the hatchery females to spawn above the dam in 2003–2005 more than doubled (2.7×) the number of second-generation recruits in the colonizing population.

On the contrary, the demographic benefit of the hatchery males was dubious. Although 55% of the assigned fathers were hatchery-produced, sex ratios were dramatically skewed toward males. There were more naturally spawned males than all females combined in 6 of 7 years (Table 1). Given the ability of males to reproduce with many females, and the tendency for salmon breeding systems to have male-biased operational sex ratios (e.g., Quinn et al. 1996), it is unlikely that any female would have failed to spawn for lack of a mate had the hatchery males been excluded from the spawning grounds. Indeed, even under experimentally altered, female-biased sex ratios of sockeye salmon, the vast majority of deposited eggs were fertilized (Mathisen 1962). It seems that cases of salmon recolonization from a source population downriver may generally experience a surplus of males. For example, sockeye salmon are prevented from spawning above Landsburg Diversion Dam, so all fish that arrive at the ladder are immigrants to the habitat above the dam or ‘strays’ from downriver. For the years 2003–2009, there was a consistent excess of male sockeye salmon (mean = 70.9%, range = 59.4–80.3%, total *N* = 6826, Seattle Public Utilities data).

Our second objective was to compare the RS of hatchery and naturally spawned colonists. Hatchery males produced fewer offspring per capita (but not significantly so) than naturally spawned fish in all 3 years, but females showed no consistent trend. Male Pacific salmon compete with each other for access to females, and females compete with each other for breeding territories (Quinn 2005). Low densities provided females with an abundance of potential spawning sites, but males probably faced fierce competition because of the skewed sex ratio. This discrepancy in the intensity of intrasexual competition could explain the relative RS patterns, and was supported by the greater opportunity for selection in males relative to females (Table 2). Consistent with this hypothesis, Fleming and Gross (1993) found that the reproductive advantage of wild over hatchery coho salmon increased more steeply in males compared to females when density, and hence competition, was experimentally increased. Similarly, Thériault et al. (2011) observed that the relative RS of hatchery males was less than that of hatchery females, and proposed that absence of sexual selection in the hatchery may be a general mechanism for fitness declines of hatchery fish.

Although the GLM did not detect a significant difference between hatchery and naturally spawned fish of either sex, the variance in RS and small sample sizes limited our statistical power. Only a very large effect of origin on RS would have been detectable with *P* < 0.05: *δ*_max_ was larger than the mean RS pooled across years for males, and three times greater than mean RS for females. As exemplified in our results, reliance on *P* < 0.05 is extremely risky in conservation biology because population sizes are often small, and accepting a false null hypothesis to guide management can have dire consequences (Taylor and Gerrodette 1993).

The comparison of RS between hatchery and naturally spawned salmon essentially tested the effects of a single generation in the hatchery. The pairwise *F*_ST_ values in our study were an order of magnitude lower than the average *F*_ST_ value of 0.033 reported by Ruckelshaus et al. (2006) across 35 subpopulations of Puget Sound Chinook salmon. Combined with the number of hatchery strays on the spawning grounds, both above (this study) and below (Berge et al. 2006) the dam, this indicates ongoing gene flow from the hatchery into the naturally spawning population. The naturally spawned and hatchery Chinook salmon clearly had similar ancestry, suggesting that the RS patterns we observed were caused by a single generation of hatchery reproduction rather than genetically based stock differences. We cannot attribute fitness differences between the two groups to specific breeding or rearing protocols because we cannot know precisely where the hatchery fish originated (i.e., Issaquah, UW, or more distant facilities). Regardless, all hatchery fish differed from naturally spawned fish in two important ways: their parents were bred artificially without natural patterns of sexual selection or mate choice, and they were reared in a controlled setting rather than a river from fertilization until their release.

Our data are consistent with the observations of Araki et al. (2008) that underperformance of hatchery fish relative to wild fish is less pronounced in systems where hatchery and wild fish have recent common ancestry. Similar to the results presented here, Ford et al. (2006) found no significant difference between hatchery and wild coho salmon where the naturally spawning population received heavy hatchery influence (range in relative RS = 0.70–1.05). Greater differences in relative RS have been demonstrated in systems with segregated, genetically distinct hatchery stocks (Araki et al. 2008). We did not expect strong genetic divergence between the naturally spawned and hatchery fish in our study because they shared a common ancestor within the last century; the Green River population provided the founding stock for both hatcheries in the basin and was historically connected to the Cedar River. However, the continued straying of hatchery fish onto the Cedar River spawning grounds above and below the dam serves to maintain genetic homogeneity (e.g., McClure et al. 2008; Barbanera et al. 2010) and this has important consequences for local adaptation (discussed further in *Implications for reintroduction programs*).

Our third objective was to evaluate the patterns of selection on body size and date of arrival to the breeding grounds. Similar to previous genetic assessments of RS, selection favored larger salmon in both sexes (Seamons et al. 2007; Anderson et al. 2010; Williamson et al. 2010). One might expect stabilizing selection to be observed in at least some studies if body size had reached an evolutionary equilibrium, so consistent selection for larger size seems paradoxical. However, fitness advantages of large animals are often masked by viability selection at other life stages (Blanckenhorn 2000). For semelparous ocean-type Chinook salmon, older fish tend to be larger, but must therefore endure additional ocean mortality risk prior to breeding that may offset the reproductive advantages of large size. Another interesting result was that selection on body size was stronger in females than males. In addition to producing more eggs than smaller females, large females have the advantages of greater egg size (Einum et al. 2004) and deeper (hence safer) egg burial (Steen and Quinn 1999); therefore, their success likely did not result from direct competition for breeding territories. With respect to males, Gross (1985) hypothesized that a sneak mating strategy by precocious ‘jack’ salmon would result in divergent selection on body size, but we did not observe this pattern in any year.

Patterns of selection on arrival date were more variable than body size. The consistent negative linear gradients indicated an advantage to early arrival, a pattern that could be explained by a prior residence advantage in competition for breeding resources (Foote 1990) or poor survival of late emerging offspring (Einum and Fleming 2000). However, it also appeared that in some years, RS was greatest at intermediate arrival dates (Fig. 4). This suggests that there may be costs to arriving too early such as encountering few potential mates or increasing the risk that offspring must begin exogenous feeding prior to increased food availability the following spring. In contrast to species with extended parental care, salmon cannot compensate for adverse conditions confronted by their offspring at hatching. Moreover, physical habitat variables affecting offspring survival (e.g., river discharge) are highly variable during the winter months when Chinook salmon typically emerge. As a result, it seems likely that offspring viability has a strong, but unpredictable, role in selection on the timing of breeding in salmon and other fishes inhabiting dynamic environments.

The measurements of selection, coupled with a comparison of trait values, allowed us to explore mechanisms for fitness differences between hatchery and naturally spawned salmon. We can reject the hypothesis that smaller size caused the hatchery males to be less productive because the hatchery males were actually larger in 2 of 3 years. Indeed, had the two groups been of equivalent size in these 2 years, the relative RS values may have been lower than what we observed. On the contrary, it seems more likely that arrival date played a role in the lower RS of hatchery males. Early arriving males had higher RS, and hatchery fish consistently arrived a few days later than naturally spawned fish (Table 4). Although these differences were small and not statistically significant, timing may have played a subtle role in competition for females or offspring survival. Interestingly, the difference in RS between hatchery and naturally spawned fish was smallest in 2005, when selection on arrival date was weakest. The later arrival timing of hatchery fish in both sexes was surprising given that salmon hatcheries, including the two in the Lake Washington basin, often select for earlier spawning dates over time (Quinn et al. 2002). This suggests that late arrival may be associated with a breakdown of olfactory homing if ‘stray’ salmon outside their natal stream migrate more slowly to their eventual spawning grounds.

All of our sampling occurred at a single location (the dam), and this resulted in some limitations that merit discussion. One surprising result was the large proportion of parentage assignments (34%) that only assigned a single parent. We sampled virtually all the salmon that ascended the fish ladder; therefore, it is implausible that the few salmon evading sampling represented all the missing parents. The most likely explanation for the father-only assignments was that some males were sampled ascending the fish ladder, but subsequently moved back downriver and spawned with unsampled females below the dam. Indeed, radio tracking revealed that such movements were common among the male coho salmon *O. kisutch* (Anderson and Quinn 2007). On the contrary, such behavior was unlikely to account for the majority of offspring assigned mothers but not fathers because spawning surveys indicated that most females ascending the fish ladder successfully constructed nests above the dam (K. Burton, Seattle Public Utilities, unpublished manuscript). Unsampled precocious male parr could account for some of the missing fathers, as was inferred in steelhead trout (Seamons et al. 2004). Mature male parr (non-anadromous individuals) are more common in interior populations of Chinook salmon that have a stream-type life history (Healey 1991). However, low densities of age-0 Chinook salmon were frequently observed in the Cedar River during summer snorkel surveys, after the majority of juveniles have migrated to the ocean (P. Kiffney, NOAA Fisheries, unpublished data). Finally, our assessment of RS did not include offspring that returned to Cedar River below the dam, or to other river systems. However, our goal was to compare the contribution of hatchery and naturally spawned fish to the next generation of colonization above the dam; therefore, it is appropriate that they were not counted.

### Implications for reintroduction programs

Our results provided evidence that the two sexes exhibited an asymmetric trade-off between demographic benefit and genetic risk of permitting the hatchery fish to colonize. For females, the demographic benefit of the hatchery fish was moderate to large, and there was no apparent fitness cost in terms of relative RS. Permitting hatchery females to bypass the dam and spawn seems to have been beneficial, as they greatly increased the number of second-generation recruits. On the contrary, the demographic benefit of allowing hatchery males to enter was negligible because males were surplus to the needs of the females, and the RS data indicated a fitness cost (10–30%) one generation after hatchery propagation. The long-term population consequences of permitting the hatchery males to breed are more difficult to assess because our study design confounded the genetic and environmental effects of captive breeding. It is possible that the hatchery males simply were less successful at acquiring mates, but did not transmit a fitness cost to their offspring relative to naturally spawned males. However, a similar study attributed the lower RS induced by hatchery ancestry to genetic effects (Araki et al. 2007), and these fitness costs carried over to the next generation of wild-born offspring (Araki et al. 2009). On the Cedar River, preventing hatchery males from reaching the spawning grounds above the dam would substantially reduce the level of artificial selection and the risk of a genetic fitness decline, likely with little or no demographic cost.

The different risk versus benefit trade-off for the two sexes emphasizes the value of females in reintroduction efforts. In this case, the colonists dispersed naturally into the newly accessible habitat, and the primary reintroduction cost was the construction, operation, and maintenance of the fish passage facilities. In contrast, reintroduction of other species typically requires active translocation (Wolf et al. 1996; Griffiths 2008), an approach that permits some control over the sex ratio of the colonist group. However, resources available for trapping, breeding, and transplanting may limit the total number of released animals. In such cases, particularly for polygynous species where only a fraction of the males successfully mate, female skewed release groups may increase the growth rate of the incipient population (Sigg et al. 2005; Lenz et al. 2007). In species whose mating system is characterized by sexual conflict, male skewed sex ratios may even hinder population growth rate (Rankin and Kokko 2007), and this would reduce the likelihood of establishing a self-sustaining population during reintroduction.

Our results suggest that natural and sexual selection have an important role in colonization. We observed selection on both of the traits we measured in this study and in a parallel investigation of coho salmon (Anderson et al. 2010). Given that large variance in RS (i.e., Fig. 2) increases the opportunity for selection (Brodie et al. 1995), there were probably other traits we did not measure that were also under selection. By benefitting fitness, adaptive evolution resulting from selection on heritable traits can increase both the likelihood of establishment and growth rate of colonizing populations (Kinnison and Hairston 2007). Indeed, many examples of contemporary adaptation are associated with introductions to new environments (Reznick and Ghalambor 2001), and invasive species often exhibit a lag time from initial colonization to population growth that might be explained by evolutionary processes required to increase fitness (Sakai et al. 2001). Selection may also shape the fitness of populations recovering from disturbance, as demonstrated by the stronger demographic contribution of locally adapted guppies compared to maladapted individuals within streams affected by catastrophic flooding (Weese et al. 2011).

Promoting natural patterns of selection therefore appears to be an important goal for reintroduction programs. Although captive breeding programs can certainly select earlier or larger animals such as those that were most successful in this study, the patterns of natural selection are impossible to emulate precisely, hence some level of domestication selection is unavoidable (Waples 1999). Moreover, adaptation to captivity can occur in a single generation (Christie et al. 2012). For reintroductions that employ captive breeding, the degree to which selection in captivity differs from the wild will ultimately govern a population's ability to adapt to its new environment.

In selecting a reintroduction strategy, programs have some control over the influence of artificial or domestication selection on colonization dynamics. In our study, captively bred animals colonized only if they dispersed into the newly accessible habitat without human assistance. Particularly for migratory species, natural selection of the founding individuals from the source population(s) may have important consequences for the adaptive evolution of the incipient population (Quinn et al. 2001); this process would be artificialized by randomly choosing individuals to actively transplant. Furthermore, reintroduction on the Cedar River maintained breeding and early life history selection in the wild. Large-scale stocking of juvenile Chinook salmon using traditional hatchery practices, an alternative scenario considered for this reintroduction, would have dramatically altered selection patterns by eliminating breeding competition, mate selection, and the influence of the river environment on early life survival of fish spawned in the new habitat. It is also possible to minimize domestication selection by selecting a source population that has been maintained in captivity for a minimal number of generations and incorporated wild individuals into the breeding protocol (Frankham 2008; Williams and Hoffman 2009).

Ultimately, these risks of an altered selection regime must be balanced against the demographic benefit provided by captive breeding during reintroduction. The benefit will be greatest initially when wild animals are unavailable or unlikely to disperse into the reintroduction site, but will decline as a colonizing population grows in abundance and becomes demographically independent. If and when this occurs, eliminating the influence of domestication selection entirely would enhance the opportunity for adaptive evolution because even low levels of gene flow from captively bred animals into the wild can shift phenotypes away from fitness maxima (Ford 2002). On the Cedar River, even the naturally spawned salmon probably already deviate from the fitness maximum as a result of a legacy of gene flow from hatcheries into the naturally reproducing population. Although the hatchery females were demographically beneficial in the early stages of colonization, curtailing hatchery immigration once the population becomes self-sustaining would maximize the probability of long-term persistence.

## Data archiving statement

Data available at Dryad repository: doi:10.5061/dryad.n4s1r
